# Melatonin Alleviates Graft Biliary Fibrosis by Inhibiting VIM^+^ Cholangiocyte Subcluster via Hypoxia/TGF‐β‐CREM‐VIM Axis

**DOI:** 10.1002/advs.75885

**Published:** 2026-06-03

**Authors:** Zhaoyi Wu, Nengsheng Fu, Zhongxin Huang, Yu Mou, Ensi Ma, Xiaoshi Wang, Tian Dong, Yujun Zhang, Danqing Liu, Di Jiang, Jingya Kuang, Rui Liao, Yifeng Tao, Leida Zhang, Chengcheng Zhang

**Affiliations:** ^1^ Department of Hepatobiliary Surgery Southwest Hospital Third Military Medical University (Army Medical University) Chongqing China; ^2^ Department of Radiology Women and Children's Hospital of Chongqing Medical University Chongqing China; ^3^ Liver Transplantation Center Department of General Surgery Huashan Hospital & Institute of Organ Transplantation Fudan University Shanghai China

**Keywords:** biliary fibrosis, CREM, epithelial‐mesenchymal transition, liver transplantation, melatonin

## Abstract

Non‐anastomotic stricture (NAS), a severe liver transplantation (LT) complication, is pathologically defined by biliary fibrosis, with unclear mechanisms and limited pharmacological therapeutic strategies. Single‐cell RNA sequencing was performed on the bile duct tissues of NAS patients, ongoing (fibrosis persistent group) or succeeding (fibrosis remission group) in endoscopic treatment. Integrated analysis indicated that biliary epithelial‐mesenchymal transition (EMT) and fibrosis were reduced in the fibrosis remission group than in the fibrosis persistent group. VIM^+^ biliary epithelial cell (BEC), a novel EMT‐related BEC subcluster, exhibited robust activation of the EMT pathway and decreased during biliary fibrosis remission. Mechanistically, extracellular hypoxia and TGF‐β signaling enhanced CREM transcriptional activity in VIM^+^ BEC, which promotes EMT by regulating the VIM expression. CREM knockdown in BEC significantly suppressed hypoxia‐ and TGF‐β‐induced VIM expression. BEC‐specific Crem knockout (Crem^fl/fl^; Sox9‐Cre^+/−^) suppressed Vim expression, EMT, and fibrosis in rats post‐LT. Melatonin administration reduced Crem expression, biliary EMT, and fibrosis in rats post‐LT. An explorative clinical trial preliminarily confirmed that some NAS patients showed improved liver function after melatonin treatment. Our findings revealed that the hypoxia/TGF‐β‐CREM‐VIM axis modulates the VIM^+^ BEC subcluster, which in turn drives biliary EMT and fibrosis, and melatonin emerges as a promising drug candidate for NAS‐related biliary fibrosis.

## Graphical abstract

Single‐cell RNA sequencing verified the presence of the VIM^+^ biliary epithelial cell (BEC) in the bile ducts of NAS patients. The hypoxia/TGF‐β‐CREM‐VIM axis mediated phenotypic switch toward VIM^+^ BEC is validated using a hypoxia/TGF‐β‐stimulated cellular model, a rat liver transplantation model, BEC‐specific Crem conditional knockout rats, and BEC organoids. Melatonin is an effective therapeutic agent.

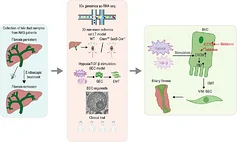

## 1 Introduction

Non‐anastomotic stricture (NAS) is the most severe postoperative complication of liver transplantation (LT) with an incidence rate of 30% [1, 2]. NAS is characterized by the stubborn and complicated biliary fibrosis on pathology and imaging [3]. Currently, the only treatment for NAS is biliary stent implantation using endoscopic retrograde cholangiopancreatography (ERCP), except for re‐transplantation. The median treatment duration is approximately 2 years and 8–10 times of in‐hospital biliary stent implantation, with a success rate of only 35%–65% [4, 5]. Thus, exploring the unclear pathophysiological mechanism of graft biliary fibrosis and therapeutic target is beneficial for shortening NAS treatment duration and improving its success rate.

Biliary fibrosis is the key feature in cholangiopathies such as NAS, biliary atresia (BA), primary biliary cholangitis, and primary sclerosing cholangitis [6, 7]. These conditions primarily affect the bile ducts, triggering fibroinflammatory reactions that first induce biliary fibrosis and then progress to bridging fibrosis and cirrhosis [8]. Notably, a proposed mechanism suggests that senescent biliary epithelial cells (BEC) persist in the bile duct and drive both the onset and persistence of peribiliary fibrosis by activating fibroblasts [9]. Prolonged injury and stimulation following LT can initiate a ductular reaction (DR), characterized by atypical biliary proliferation without distinct lumens [10]. This DR, along with severe bridging fibrosis, was observed in liver biopsies of 25% of the LT recipients [11]. Under physiological conditions, BEC participate in the construction of the scaffold matrix through the epithelial‐mesenchymal transition (EMT) pathway [12]. In BA, BEC undergo EMT under the stimulation of transforming growth factor (TGF), which promotes tissue fibrosis [7]. Hypoxia, a common factor in LT, induces the expression of pro‐fibrogenic factors such as vascular endothelial growth factor‐A (VEGF‐A), TGFβ1, and sonic hedgehog in the BEC [13, 14]. These factors can activate the EMT in BEC, leading to impaired restructuring of the biliary structure [8]. As a cellular phenomenon contributing to tissue fibrosis and also a component of the DR, whether EMT promotes biliary fibrosis in NAS needs investigation [11]. Previous studies have reported that cyclic adenosine monophosphate (cAMP) response element‐binding modulator (CREM) overexpression promotes atrial fibrosis, while inhibition of CREM family activity effectively attenuates renal EMT and fibrosis [15, 16, 17]. Previous studies have demonstrated that melatonin upregulates the expression of inducible cAMP early repressor (Icer) in the rat pineal gland, which in turn suppresses Crem expression [18, 19, 20]. Thus, the role of the transcription factor CREM in biliary EMT and melatonin's potential therapeutic effect on graft fibrosis via the ICER‐CREM axis warrants further investigation.

Remission of radiological features such as stricture or rigidity is a vital criterion for endoscopic treatment success and biliary stent removal [21]. Pathological fibrosis remission (FR) is essential, but the mechanism remains unclear. In this study, NAS patients were stratified into two groups: patients undergoing endoscopic treatment (fibrosis persistent (FP) group) and those who succeeded in endoscopic treatment (FR group). Liver function tests, pathological analysis, and imaging evaluations were conducted to compare the biliary fibrosis and structure between the two groups. By performing biliary biopsies via ERCP followed by single‐cell RNA sequencing (scRNA‐seq) analysis in them, we uncovered heterogeneous BEC subclusters and the molecular mechanisms driving EMT in BEC. Mechanistically, we identified the key transcription factors that mediate BEC EMT, and verified their downstream targets via chromatin immunoprecipitation (ChIP). These were validated by in vitro stimulation and in vivo LT models, and BEC‐specific conditional knockout (CKO) models were constructed to confirm the targets’ regulatory role of the targets in BEC EMT. Furthermore, we identified potential therapeutic drugs and verified their efficacy through in vitro and in vivo experiments and clinical trials. Our research delves deeper into the phenotypic transformation of BEC in graft biliary fibrosis, offering a potential therapeutic strategy.

## 2 Materials and Methods

### 2.1 NAS Patients’ Samples

The method of NAS patients’ biliary tissue acquisition was described previously [5]. The clinical characteristics were listed in Table S1. Collecting human NAS samples was approved by the Ethics Committee of the Southwest Hospital, Third Military Medical University, Chongqing, China (KY2023016).

### 2.2 Animals

Male and Female 6–8 weeks old Sprague‐Dawley rats (200–220 g) and immunodeficient NSG mice, which lack B, T, and NK lymphocytes, were used. Animals were maintained on a standard laboratory diet according to the Institutional Animal Care and Use Committee of the Army Medical University (AMUWEC20242047). All experiments were approved by the Animal Ethics Committee.

### 2.3 Culture of Primary Human Extrahepatic BEC (Hehbec)

Human primary BEC were purchased from Procell (Wuhan, China). The cells were cultured in 1640 supplemented with 20% FBS and immortalized using a lentivirus expressing SV40 largeT antigen (Genomeditech, Shanghai, China). All cultures were maintained at 37°C in a humidified atmosphere containing 5% CO2.

### 2.4 Generation and Culture of Biliary Organoids

Excised bile duct segments were rinsed once with washing buffer (D2E‐100, JKMed, Chongqing, China) in a 10‑cm dish. Each duct was incised longitudinally to expose the lumen, which was then covered with 10 mL washing buffer. The primary cholangiocytes were gently scraped using a surgical blade under rinsing buffer (D2B‐100, JKMed), and the resulting cell suspension was collected. The tissue and dish were washed 2–3 times with rinsing buffer to recover residual cells. All supernatants were combined and centrifuged at 444 g for 4 min. The cell pellet was washed and resuspended again after centrifugation. Isolated primary cholangiocytes were centrifuged at 444 g for 4 min and resuspended in Matrigel (D2H‐10, JKMed) mixture. Part of the primary cholangiocytes were subjected to MagniSort Dynabeads sorting to isolate MUC6^+^ cholangiocytes. The acquired cholangiocytes were further cultured in the BEC organoid culture system (E‐240118, JKMed).

### 2.5 Clinical Study Design and Study Population

To evaluate the efficacy of melatonin in NAS patients, we proposed an exploratory trial without randomization or blinding. Melatonin (10 mg) was orally administered every night for 8 weeks. Baseline data were collected at enrollment.

The primary endpoint was the alteration in biliary enzymes (gamma‐glutamyl transpeptidase (GGT) and alkaline phosphatase (ALP)). The secondary endpoints included bilirubin, alanine transaminase (ALT), aspartate aminotransferase (AST), and adverse index (Pittsburgh Sleep Quality Index (PSQI) scales and Treatment Emergent Symptom Scale (TESS)). Liver function tests were performed at weeks 1, 2, 3, 4, 6, and 8. The PSQI and TESS were used to evaluate adverse effects and sleep quality, respectively. The two scales were recorded by the participants at weeks 4 and 8. Liver function and scales were assessed before enrollment.

Patients who met the following criteria were enrolled: (1) 18–70 years; (2) biliary complications were clearly diagnosed by ERCP after liver transplantation and were treated with endoscopic biliary stent implantation; (3) relatively stable liver function (in the two liver function results with an interval of more than 2 weeks, the fluctuation of biliary enzyme spectrum ALP and GGT less than 20%), and the patients with stents had >2 weeks interval after the last implantation, or stent free patients >2 weeks interval after stent removal; (4) abnormal biliary enzyme (ALP or GGT greater than 1.5 times the upper limit); (5) abnormal liver function not caused by other reasons.

This clinical study was approved by the Ethics Committee of Southwest Hospital (KY2024141) and registered at Chictr.org (https://www.chictr.org.cn, ChiCTR2400090706). This trial was conducted in accordance with the Declaration of Helsinki, and informed consent was obtained from all participants before enrollment. All documents, variables, and adverse events were recorded in an Electronic Data Capture (EDC) system in a timely manner.

### 2.6 Statistical Analysis

Quantitative data were presented as mean ± standard deviation, median (IQR), or frequency, as appropriate. Data were presented as medians using box plots and as means using bar plots. Statistical analysis and graphical representation of the data were performed using R (version 4.2.3). Comparisons between two groups were performed using the Wilcoxon rank‑sum test for non‑normally distributed continuous data, two‐tailed paired or unpaired Student's *t* tests for normally distributed data, and the chi‑square test or Fisher's exact test for categorical variables. Statistical significance was tested using two‐tailed paired or unpaired Student's t‐test (ns, no significance; ^*^ *p* < 0.05; ^**^ *p* < 0.01; ^***^ *p* < 0.001).

### 2.7 Others

Please refer to the Supporting Information.

## 3 Results

### 3.1 Biliary FR in NAS is the Core Feature of Successful Multiple Endoscopic Treatments

**FIGURE 1:**
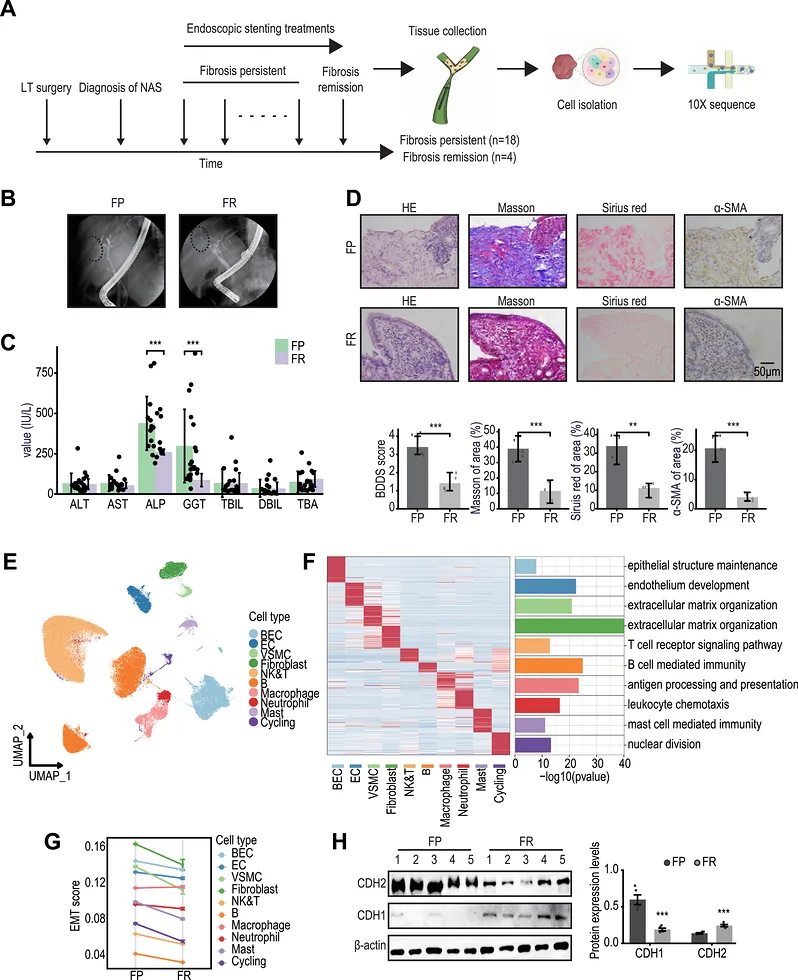
Bile duct EMT tendency and FR post‐successful endoscopic treatment. (A) Schematic diagram of tissue collection from NAS patients in the FP and FR groups. (B) Cholangiographic imaging reveals the changes in biliary structure in NAS patients between the FP and FR groups. (C) The change of liver function (ALT, AST, ALP, GGT, TBIL, DBIL, and TBA) between the FP and FR groups. (D) HE, Masson, Sirius red, and α‐SMA staining of representative bile ducts in the FP and the FR groups. The BDDS scores and fibrosis staining area were compared between the two groups. Scale bars, 50 µm. (E) UMAP plots showing the distribution of different cell types in the bile duct. (F) Heatmap showing the specific genes and function of each cell type. (G) Line graph showing the EMT scores of various cell populations between two groups. (H) Protein expression levels of CDH1 and CDH2 in bile ducts from the two groups were detected by WB. DBIL, direct bilirubin.

To investigate the mechanism of biliary fibrosis changes during endoscopic treatment, we collected bile duct tissues via ERCP from the FP and FR groups (Figure 1A). Biliary tree morphology was visualized by cholangiography (Figure 1B), and the severity of stricture was graded according to the criteria described by Buis et al. [22], revealing significantly higher biliary stricture severity in the FR group than in the FP group (Table S1). NAS patients exhibited gradual liver function remission during multiple treatments (Figure S1A). Compared with the FP group, liver function parameters, especially GGT and ALP, biomarkers of cholangiopathies [23], were significantly reduced in the FR group (Figure 1C). Biliary samples from the FR group manifested a more continuous BEC structure with a lower bile duct damage score (BDDS) score and less fibrosis area in staining (Figure 1D) [24].

### 3.2 Single‐Cell Transcriptional Profiling of Downregulated EMT Potential During FR

To investigate transcriptional changes in biliary tissues during FR, we conducted scRNA‐seq on bile ducts from 22 NAS patients (FP: 18; FR: 4). After quality control (Figure S2A), a total of 151,509 cells were sequenced (Figure S2B) and formed 10 main cell types according to cellular marker genes and functions: BEC, endothelial cell (EC), vascular smooth muscle cell (VSMC), fibroblast, NK&T, B, macrophage, neutrophil, mast, and cycling cell (Figure 1E, F).

The contribution and cellular proportion of per‐patient were shown in Figure S2E, F. Consistent with the pathological findings, a restoration of the BEC proportion and a significant reduction in fibroblast were observed during FR (Figure S2G, H). This trend was consistent with the EMT process [25], and the EMT pathway was activated in BEC and fibroblast (Figure S3B). The EMT score was reduced (Figure S3C) while the scores of EMT in each cell type were reduced during FR in which BEC and fibroblast showed the highest EMT scores (Figure 1G). Western blot (WB) and immunohistochemistry (IHC) showed increased CDH1 and decreased CDH2 expression during FR (Figure 1H; Figure S3D).

### 3.3 Single‐Cell Transcriptomics Identifies a Novel EMT‐Related VIM^+^ BEC Subcluster

**FIGURE 2:**
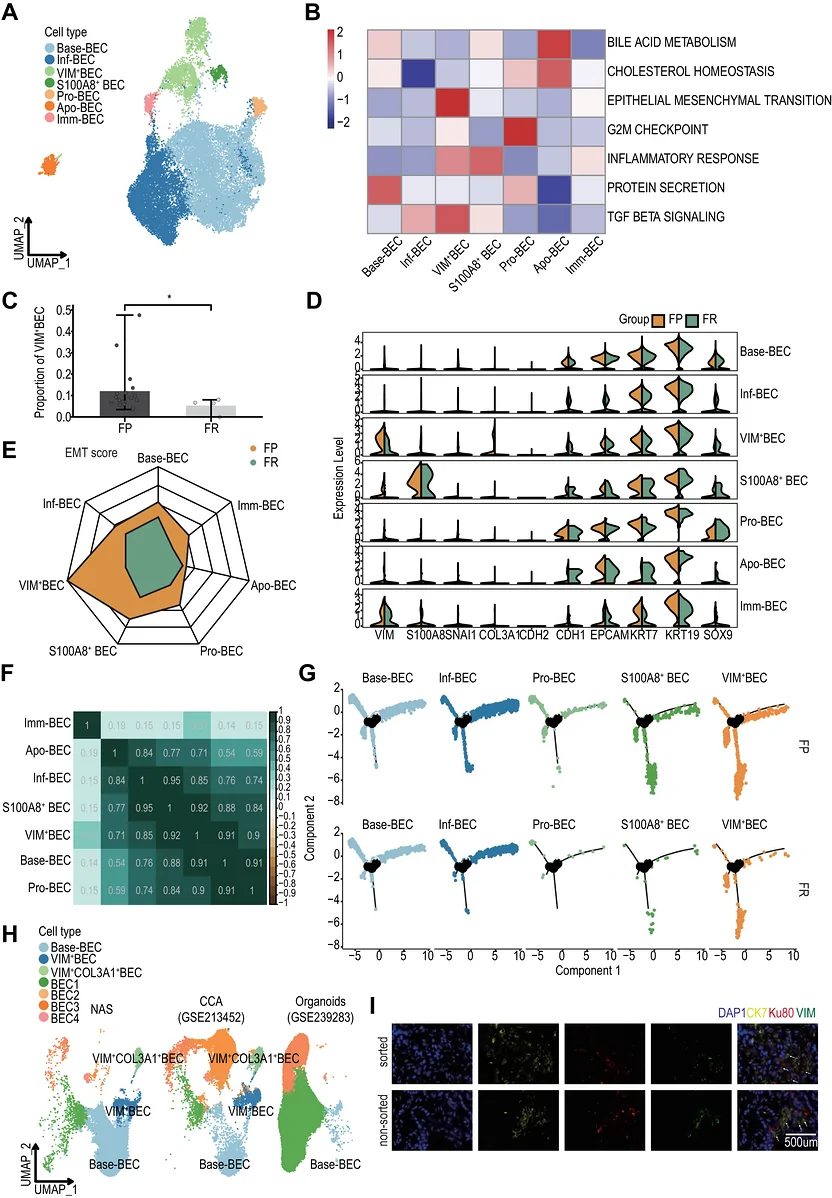
VIM^+^ BEC subcluster occurring during biliary fibrosis. (A) UMAP plots showing the distribution of different BEC subclusters. (B) Heatmap showing the function of each BEC subcluster. (C) Bar plots showing the proportion of VIM^+^ BEC between the FP and FR groups. (D) Violin plots showing the expression of EMT‐related genes in distinct BEC subclusters between the two groups. (E) Radar plots showing the EMT pathway scores of various BEC subclusters in the two groups. (F) The heatmap illustrates the correlations among various BEC subclusters. (G) Pseudo‐trajectory of five BEC subclusters in the two groups. (H) UMAP plots showing the distribution of BEC subclusters among NAS (our data), CCA (GSE213452), and organoid (GSE239283) groups. (I) IF images of bile ducts after biliary organoid transplantation: Ku80‐positive cells represent human‐derived cells, which were present in the biliary tissue following organoid engraftment. Ku80^+^VIM^+^ cells were integrated in the tissues of both the sorted and non‐sorted groups (white arrows). Scale bars, 500 µm.

To validate the subcluster composition and the mechanism of EMT in BEC, we divided BEC into seven subclusters (Figure 2A). A novel EMT‐related BEC subcluster was identified: VIM^+^ BEC (VIM, COL1A1, COL3A1) with the highest EMT pathway scores(Figure 2B) [12]. VIM^+^ BEC clusters showed a decreased proportion during FR (Figure 2C; Figure S4A–D). The remaining clusters were: peribiliary glands (PBG), base BEC (Base‐BEC), inflammatory BEC (Inf‐BEC), S100A8^+^ BEC, lipid‐associated BEC (Lip‐BEC), immune‐associated BEC (Imm‐BEC), and proliferative BEC (Pro‐BEC) [26, 27, 28].

Inhibition of VIM effectively improves tissue fibrosis [29, 30]. UMAP showed a denser distribution of VIM in the BEC of the FP group than in the FR group (Figure S4E). The area with VIM expression is increased while the expression of KRT7 is decreased (Figure S4F). VIM^+^ BEC exhibited lower VIM expression levels during FR (Figure S4G). The FP group showed higher expression of mesenchymal markers, whereas the FR group demonstrated higher expression of epithelial markers (Figure 2D). The EMT pathway scores of each BEC subcluster were reduced during FR (Figure 2E).

Base‐BEC positively correlated with VIM^+^ BEC, S100A8^+^ BEC, Inf‐BEC, and Pro‐BEC (Figure 2F). Additionally, differential trajectories among these five clusters demonstrated that Base‐BEC have the potential to differentiate into the VIM^+^ BEC (Figure 2G and Figure S4H). The FP group had a higher proportion of BEC transitioning toward State 3 differentiation than the FR group (Figure 2G). State 3 had a higher expression level of VIM and a lower level of KRT19 and SOX9 than State 1 (Figure S4I). To verify the existence of the VIM^+^ BEC subcluster, we performed an integrated analysis of BEC from cholangiocarcinoma (CCA) (GSE213452), biliary organoids (GSE239283), and our NAS scRNA‐seq data (Figure S5A). We analyzed BEC subclusters, including Base‑BEC and VIM^+^ BEC (Figure S5B). VIM^+^ BEC were exclusively present in bile duct tissues from CCA and NAS patients (Figure 2H and Figure S5C). However, VIM^+^ BEC were absent, and only a small number of Base‐BEC were observed in BEC organoids established from biliary brushings obtained during ERCP, a method that poorly accesses BEC from the PBG region (Figure 2H and Figure S5C). These findings suggest the presence of VIM^+^ BEC, and they may be derived from the differentiation of Base‐BEC.

Previous studies employed BEC organoids to investigate the EMT process of BEC [12]. MUC6 serves as a marker for PBG BEC (Base‐BEC) [26]. We isolated BEC from human extrahepatic biliary tissue and sorted MUC6^+^ BEC for organoid establishment (Figure S6A). Organoids constructed from both the sorted and non‑sorted groups highly expressed MUC6 and CK7 but not VIM (Figure S6B). BEC organoids were injected into immunodeficient mice via the gallbladder. After 7 days, a population of Ku80^+^VIM^+^ cells was observed in both the sorted group and the non‐sorted group, which further verified the existence of the EMT phenomenon in PBG‐derived BEC (Figure 2I).

### 3.4 CREM Acts as an Active Transcriptional Regulator of VIM in VIM^+^ BEC

**FIGURE 3:**
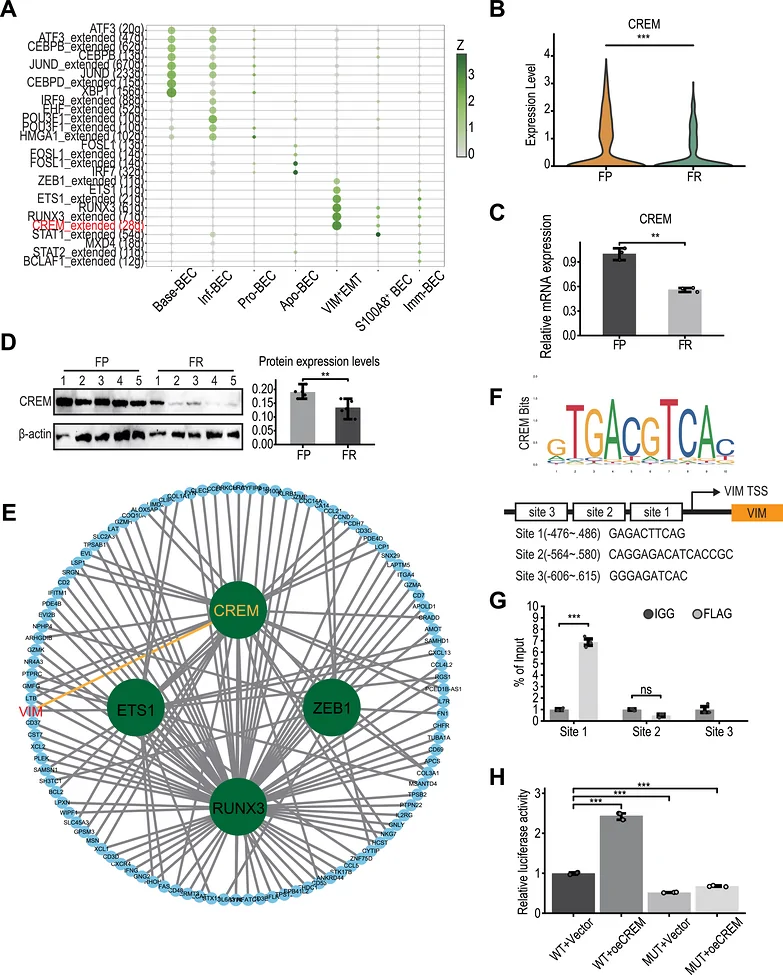
CREM promotes the transformation of VIM into VIM^+^ BEC. (A) Dot plots showing the activated transcription factors in various BEC subclusters. (B) Violin plots showing the CREM expression of VIM^+^ BEC between the two groups. (C) RT‐qPCR analysis of CREM in the two groups. (D) Protein expression levels of CREM in bile ducts from the two groups were detected by WB. (E) Transcription factor‐target regulatory network in VIM^+^ BEC. (F) The motif of CREM from the Jasper database (above). A schematic of the human VIM promoter highlighting the CREM site (below). (G) A ChIP‐qPCR assay was performed on 293T cells to quantify the physical binding of CREM to the VIM promoter region. (H) 293T cells were cotransfected with pGPL4‐VIM‐Promoter (WT or Mut) and pGPL4‐RL plasmids, plus pcDNA3.1 or CREM expression vector. Luciferase activity was measured 48 h post‐transfection.

SCENIC analysis was used to identify the master regulators driving the phenotypic transitions of VIM^+^ BEC [31]. CREM regulatory elements were active in VIM^+^ BEC (Figure 3A). VIM^+^ BEC exhibited decreased CREM expression during FR (Figure 3B). Clinical sample quantification showed that CREM mRNA and protein expression levels in the FP group were higher than those in the FR group (Figure 3C, D).

The transcription factor networks indicated that CREM regulated the expression of VIM in VIM^+^ BEC (Figure 3E). CREM expression positively correlated with EMT markers (SRGN, CSCR4, and COL6A2) in VIM^+^ BEC (Figure S7A). UMAP showed regions with higher CREM expression had higher VIM expression (Figure S7B). JASPAR database prediction revealed that CREM binds to VIM via three potential sites (Figure 3F). ChIP was conducted to validate the physical interaction of CREM with site 1 of VIM promoter region (Figure 3G). To confirm CREM as a transcriptional regulator of VIM, we performed the dual‐luciferase reporter assays. Luciferase activity was significantly increased upon co‐transfection of CREM and VIM‐WT, whereas a significant decrease was observed after mutation at site 1 of VIM and co‐transfection of CREM and VIM‐MUT (Figure 3H). Collectively, our findings demonstrate that CREM promotes VIM transcription by binding to the site 1 region of the VIM promoter.

### 3.5 Hypoxia and TGF‐β Act as Major Extracellular Signals to Upregulate CREM in VIM^+^ BEC

**FIGURE 4:**
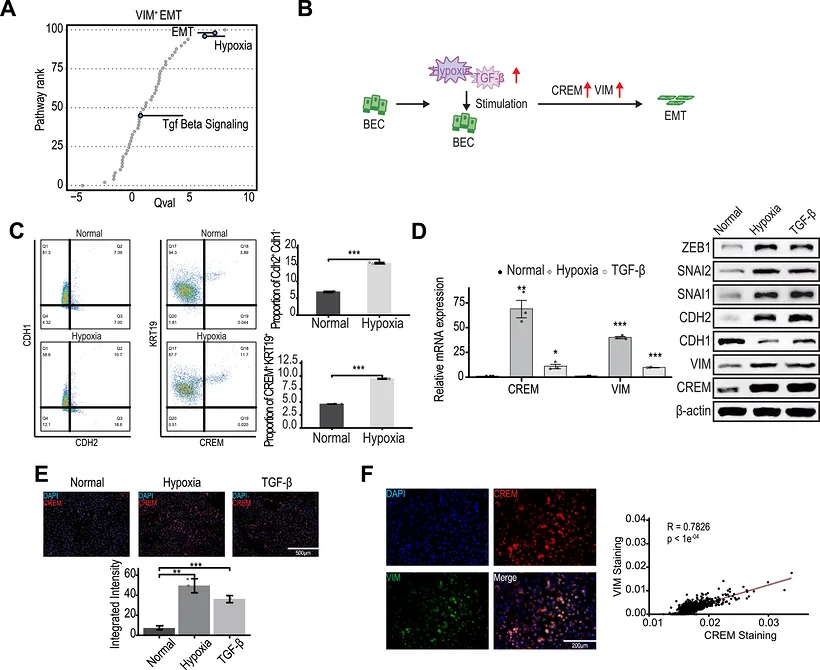
Hypoxia and TGF‐β stimulation act upstream of CREM, leading to the EMT process in BEC. (A) A dot plot showing that the EMT, hypoxia, and TGF‐β pathways were more highly expressed in the FP group compared to the FR group. (B) Schematic diagram illustrating a role for hypoxia and the TGF‐β‐CREM axis in the phenotypic switch of BEC. (C) Flow cytometry analysis of the proportion of CDH2^+^ CDH1^−^ cells and CREM^+^ KRT19^+^ cells before and after hypoxia and TGF‐β stimulation. (D) The mRNA levels of CREM and VIM and protein levels of CREM, VIM, CDH1, CDH2, SNAI1, SNAI2, and ZEB1 in HEHBEC from the normal, hypoxia, and TGF‐β groups. (E) IF staining showing the expression of CREM in HEHBEC before and after hypoxia and TGF‐β stimulation (above). Bar charts showing the integrated fluorescence intensity of CREM in the normal, hypoxia, and TGF‑β groups (below). (F) IF showing the expression of CREM and VIM in HEHBEC after hypoxia stimulation (left), and the correlation between VIM and CREM expression (right).

To clarify the mechanisms underlying CREM activation in VIM^+^ BEC, we explored the upstream extracellular signaling pathways. Gene Ontology (GO) enrichment analysis indicated the upregulation of the hypoxia and TGF‐β pathways within the VIM^+^ BEC (Figure S8A). Compared with core pathway activities, the VIM^+^ BEC of the FP group exhibited notable upregulation of the EMT, hypoxia, and TGF‐β pathways compared to the FR group (Figure 4A). The expression of hypoxia‐related genes in BEC subclusters was higher in the FP group, which was co‐expressed with CREM (Figure S8B, C).

EMT cell models were constructed using hypoxia and TGF‐β stimulation in HEHBEC (Figure 4B and Figure S8D) [32]. Microscopic examination revealed that HEHBEC exhibited a spindle fibroblast‐like morphology following hypoxia and TGF‐β stimulation (Figure S8E). Flow cytometry analysis revealed an increase in the proportion of CDH2^+^CDH1^−^ cells following hypoxia stimulation (Figure 4C). The mRNA and protein levels of CREM and VIM in HEHBEC were increased after stimulation with hypoxia and TGF‐β (Figure 4C–E). WB demonstrated a decrease in CDH1 and an increase in CREM, VIM, CDH2, SNAI1, SNAI2, and ZEB1 in HEHBEC following hypoxia and TGF‐β stimulation (Figure 4D). Linear regression analysis revealed a strong positive correlation between CREM and VIM expression levels (Figure 4F).

### 3.6 Upregulation of the Hypoxia/TGF‐β‐Crem‐Vim Axis is Observed in Rat Biliary Grafts Post‐LT with Warm Ischemic Injury

**FIGURE 5:**
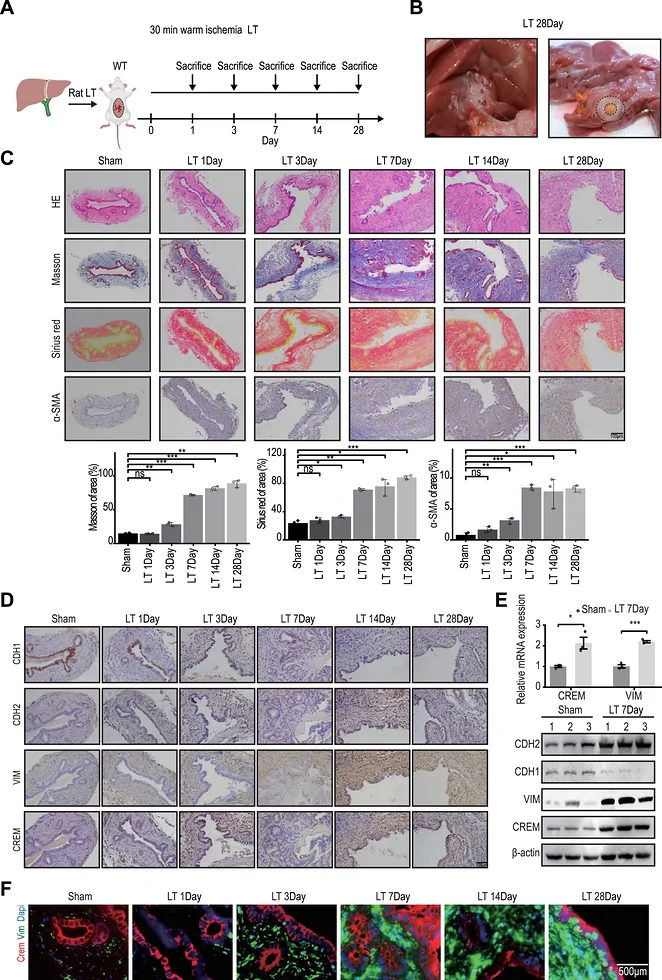
Hypoxia/TGF‐β‐Crem‐Vim axis was upregulated in rat biliary grafts post‐LT with warm ischemic injury. (A) Schematic diagram illustrating the process of rat LT used to simulate the in vivo hypoxic process of grafts. (B) At 28 days post‐LT in rats, the bile duct walls thickened, and bile accumulation was observed within the duct walls. (C) HE, Masson, Sirius red, and α‐SMA staining of bile ducts at 1, 3, 7, 14, and 28 days in rats post‐LT. And the fibrosis staining area was quantified in each group. Scale bars, 100 µm. (D) IHC staining showing the expression of Cdh1, Cdh2, Vim, and Crem in bile duct tissue at 1, 3, 7, 14, and 28 days in rats post‐LT. Scale bars, 100 µm. (E) The mRNA levels of Crem and Vim and protein expression levels of Crem, Vim, Cdh1, and Cdh2 in bile ducts from sham and LT 7day groups. (F) IF staining of Ck7, Vim, and DAPI in rat bile duct tissues. Scale bars, 500 µm.

Given the higher NAS incidence in donation after circulatory death graft recipients, a rat LT model with 30 min of warm ischemia was established to simulate the graft biliary hypoxia and fibrosis (Figure 5A) [33, 34]. Post‐LT, serum levels of ALT, AST, ALP, total bile acid (TBA), as well as IL‑1, IL‑6, IL‑10, and TNF‑α were all significantly elevated (Figure S9A, B). On day 28, the bile duct walls thickened, and bile accumulation was observed within the lumens (Figure 5B). The bile duct lumens gradually lost their normal morphology post‐LT (Figure 5C). Concurrently, fibrosis staining results indicated significant fibrosis on day 7 (Figure 5C). Our prior studies conducted scRNA‐seq on bile duct tissues from rats at 1, 3, and 7 days post‐LT (GSE291336), revealing significant upregulation of the EMT pathway and Crem within the bile duct tissues post‐LT (Figure S9C). Additionally, the hypoxia and the TGF‐β pathway in BEC were markedly upregulated post‐LT (Figure S9D). Cdh1 expression gradually decreased, while Crem, Vim, and Cdh2 expression significantly increased post‐LT (Figure 5D and Figure S9E). On day 7 post‐LT, the mRNA and protein expression levels of Crem and Vim were higher than those of the sham group (Figure 5E). In the bile duct tissues of rats post‐LT, Vim expression was increased in the Ck7^+^ regions (Figure 5F).

### 3.7 BEC‐Specific Crem CKO Inhibited Hypoxia/TGF‐β‐Crem‐Vim Axis in Rats Post‐LT

**FIGURE 6:**
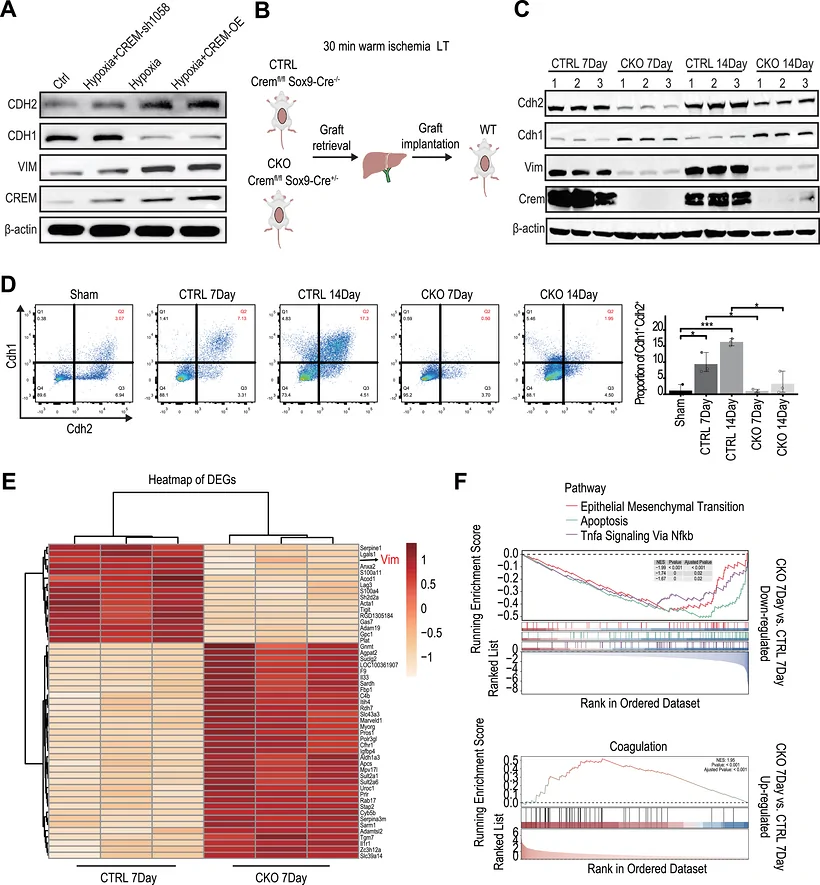
BEC‐specific Crem CKO inhibited BEC EMT and biliary fibrosis. (A) Protein expression levels of CREM, VIM, CDH1, and CDH2 in HEHBEC under different treatment conditions were detected by WB. (B) Schematic diagram of LT using BEC‐specific Crem CKO rats as donors. (C) Protein expression levels of Crem, Vim, Cdh1, and Cdh2 in two groups were detected by WB. (D) Flow cytometric analysis of Cdh1 and Cdh2 in bile duct tissues of rats post‐LT (left). Bar plots showing the proportion of Cdh1^+^Cdh2^+^ cells in different groups (right). (E) Heatmap showing the DEGs identified between two groups. (F) GSEA enrichment plot showing the up‐regulated and down‐regulated pathways between the two groups.

CREM expression in HEHBEC was knocked down and overexpressed via lentivirus (Figure S10A, B). CREM Knockdown in HEHBEC effectively inhibited the increase in VIM and CDH2 expression and the decrease of CDH1 expression in HEHBEC after hypoxia stimulation (Figure 6A). CREM overexpression had the opposite effect (Figure 6A).

To determine the function of Crem in biliary fibrosis, we investigated the phenotype of BEC‐specific Crem CKO rats (Crem^fl/fl^; Sox9‐Cre^+/−^). The knockdown efficiency of Crem was confirmed by DNA sequencing in Crem^fl/fl^; Sox9‐Cre^+/−^ rats (Figure S10C). The BEC‐specific Crem CKO rats were born without any apparent abnormalities. The liver tissues of Crem^fl/fl^; Sox9‐Cre^−/−^ (control (CTRL) group) and Crem^fl/fl^; Sox9‐Cre^+/−^ (CKO group) rats were implanted into wild type (WT) rats (Figure 6B). On days 7 and 14 post‐LT, Crem, Vim, and Cdh2 protein expression decreased in the CKO group compared with that in the CTRL group, whereas Cdh1 increased (Figure 6C). Flow cytometry analysis revealed that the CTRL group had a higher proportion of Cdh1^+^Cdh2^+^ cells than the CKO group on days 7 and 14 post‐LT (Figure 6D). In the CKO group, Vim expression in Ck7^+^ areas was lower than that in the CTRL group (Figure S11A), and the degree of biliary EMT and fibrosis was lower in the CKO group (Figure S11B).

Transcriptome sequencing was performed on the grafts of the CTRL and CKO groups (n = 3 per group) on days 7 post‐LT to identify differential expression genes (DEGs) associated with Crem deletion. Principal component analysis (PCA) showed clear separation between the two groups (Figure S11C). DEGs were identified between the two groups (Figure 6E). Among these, Vim was significantly downregulated in the CKO group (Figure 6E and Figure S11D). Gene Set Enrichment Analysis (GSEA) analysis revealed that down‐regulated DEGs of the CKO group were primarily enriched in the EMT pathway (Figure 6F).

### 3.8 Melatonin Regulates Hypoxia/TGF‐β‐Crem‐Vim Axis in Rats Post‐LT

**FIGURE 7:**
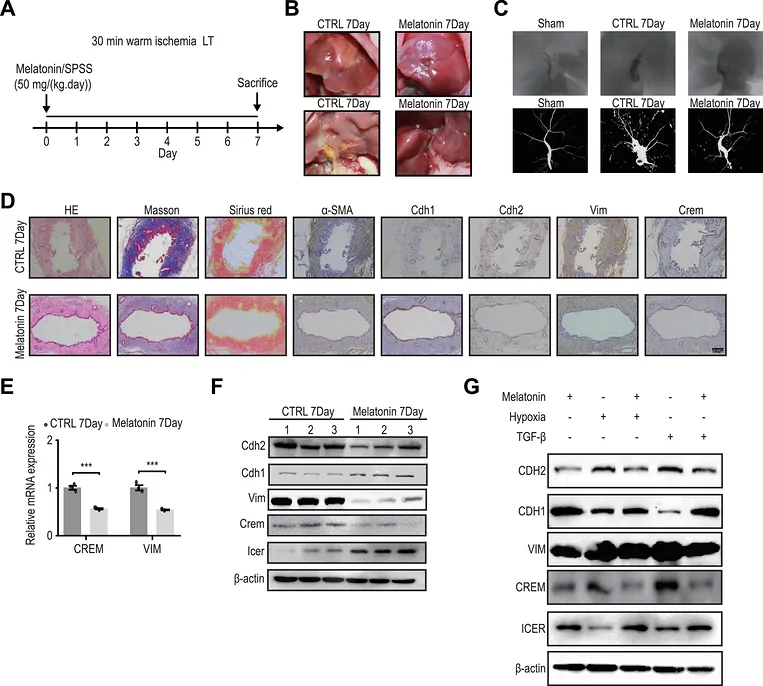
Melatonin regulates the expression of CREM and the process of EMT in BEC. (A) Schematic diagram of melatonin treatment in rat LT. The rats were divided into the CTRL group and the melatonin group according to melatonin administration, and all were sacrificed on day 7 post‐LT to collect biological samples. (B) Gross presentation of bile ducts on day 7 post‐LT in both the CTRL and melatonin groups. (C) Micro CT images showed the bile duct tree morphology in the CTRL and melatonin groups. Top, before bile duct reconstruction; Bottom, after bile duct reconstruction. (D) HE, Masson, Sirius Red, α‐SMA, Cdh1, Cdh2, Vim, and Crem staining of bile ducts in the CTRL group and the melatonin groups. Scale bars, 100 µm. (E) The mRNA levels of Crem and Vim of the bile ducts in the CTRL and melatonin groups. (F) Protein expression levels of Icer, Crem, Vim, Cdh1, and Cdh2 of bile ducts in the CTRL and melatonin groups. (G) Protein expression levels of ICER, CREM, VIM, CDH1, and CDH2 in HEHBEC under different treatment conditions were detected by WB.

Previous studies have confirmed that melatonin can promote the expression of Icer in the rat pineal gland, thereby inhibiting the expression of Crem [18, 19, 20]. By intraperitoneally injecting melatonin at a dose of 50 mg/(kg.day) in post‐LT rats, the remission of biliary EMT and fibrosis was achieved (Figure 7A). Gross appearance revealed that on the 7 day post‐LT, the graft of the CTRL group exhibited significant bile accumulation, with partial obstruction and necrosis in some areas, whereas the melatonin group maintained a more normal state of the graft (Figure 7B). Micro CT reconstruction and cholangiography of the biliary tree showed deformities in the CTRL group, with surrounding bile duct stricture and continuity disruption, while the melatonin group maintained a more continuous morphology (Figure 7C). Pathological staining indicated a reduction in biliary EMT and fibrosis (Figure 7D and Figure S12A). The ALP and TBA levels were significantly lower in the melatonin group (Figure S12C). Post‐LT, mRNA levels of Crem and Vim were lower in the melatonin group. WB results indicated increased levels of Cdh1 and Icer, and decreased levels of Crem, Vim, and Cdh2 in the melatonin group (Figure 7F).

In vitro, melatonin effectively promoted the expression of ICER and inhibited the expression of CREM and VIM in HEHBEC following hypoxia and TGF‐β stimulation (Figure 7G and Figure S12D). This, in turn, ameliorated EMT in HEHBEC after hypoxia and TGF‐β stimulation (Figure 7G).

### 3.9 Therapeutic Effect of Melatonin in NAS Patients

**FIGURE 8:**
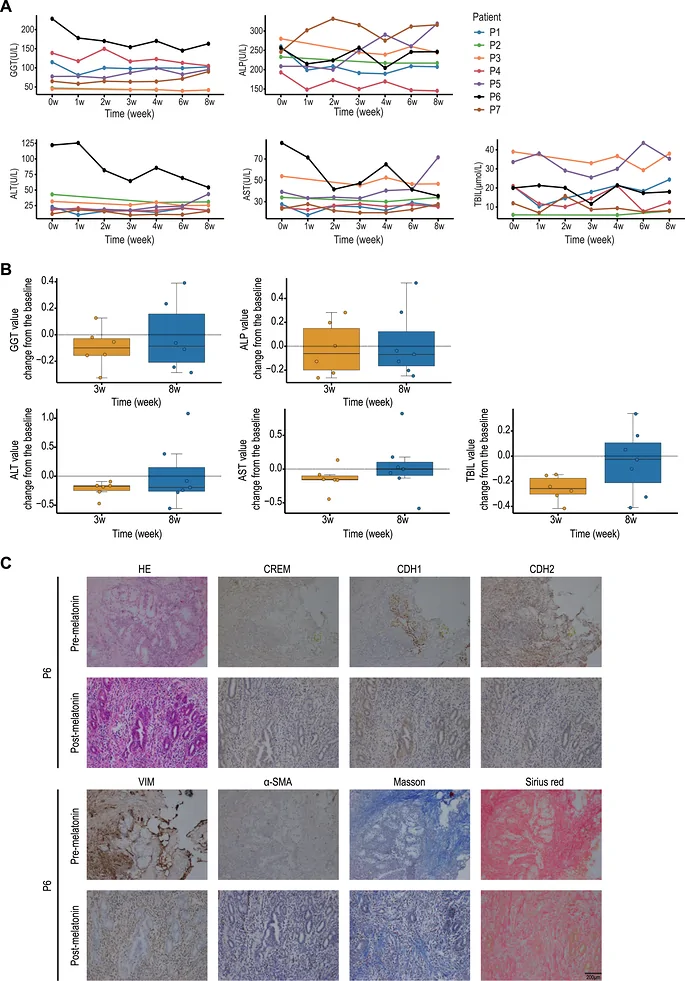
Improvement of liver function of NAS patients after oral melatonin administration. (A) Chronological changes in the liver function for each patient from registration (0 week) to the end of the observation period (8 weeks). (B) Changes in liver function at 3 and 8 weeks compared with the baseline. (C) HE, CREM, CDH1, CDH2, VIM, α‐SMA, Masson, and Sirius red staining of the bile ducts of patient 6 pre‐ and post‐oral melatonin. Scale bars, 200 µm.

The baseline characteristics of the seven patients were shown (Table S2). The median disease duration of the seven NAS patients was 196 days, and the median endoscopic treatment duration was 983 days. Serum levels of GGT, ALP, ALT, and AST showed an overall decreasing trend (Figure 8A). A mild elevation of ALP was observed in patient P7, while ALP and AST increased after 3 weeks of treatment in patient P5 (Figure 8A). Serum total bilirubin (TBIL) remained relatively stable with no obvious fluctuation (Figure 8A). Improvement in liver function indicators occurred rapidly within the first 3 weeks of medication, followed by a relatively stable phase from week 3 to week 8 (Figure 8B). At week 8, GGT and ALP in P4 decreased by 24%. In P6, GGT decreased by 28%, ALT decreased by 56%, and AST decreased by 58%. Bile tissues from patient P6 manifested a more intact morphology, with a decreasing trend in the CREM expression and the degree of EMT and fibrosis (Figure 8C) after melatonin treatment. The PSQI scales and TESS scales were used to assess the safety of NAS patients pre‐ and post‐taking melatonin, which confirmed that oral melatonin had no safety‐related effects on NAS patients (Figure S13A).

## 4 Discussion

FR is accompanied by more patent bile duct lumens, improved liver function parameters, reduced degrees of EMT, and fibrosis in the bile ducts. NAS, a long‐term complication post‐LT, is characterized by a prolonged pathogenesis and treatment cycle [2, 35]. Long‐term injury and stimulation triggered DR [10]. As a part of DR, EMT was a cellular phenomenon that leads to tissue fibrosis [36]. Biliary fibrosis is commonly seen in cholangiopathies [3, 37, 38]. Its core lies in the abnormal initiation of repair following injury to BEC, and EMT serves as the key mechanism by which BEC contribute to fibrosis [39]. EMT occurs in BEC following injury, and inhibition of EMT in BEC can effectively improve BA [7, 12]. Similarly, our scRNA‐seq indicated that BEC exhibit extremely high EMT activity during NAS. We detected VIM^+^ BEC, a novel EMT‐related BEC subcluster, and further confirmed its presence in CCA. Compared with other BEC subclusters, this VIM^+^ BEC exhibits a higher level of VIM expression and EMT pathway activation. In contrast, the proportion of VIM^+^ BEC showed a decreasing trend during the FR, accompanied by a reduction in the activity level of the EMT pathway. Inhibition of VIM expression effectively alleviates tissue fibrosis [29, 30], suggesting that suppressing the transition of VIM^+^ BEC may mitigate biliary EMT and fibrosis.

We preliminarily explored the origin of VIM^+^ BEC. Correlation and pseudotime analyses suggested that VIM^+^ BEC might derive from PBG BEC (Base‐BEC). To verify this hypothesis, we integrated our scRNA‐seq data with those of CCA and BEC organoids [40]. VIM^+^ BEC were present in biliary tissues under NAS and CCA pathological conditions, but absent in luminal BEC‐derived organoids obtained by ERCP. PBG BEC mainly localizes in deep PBG regions, which are hardly accessible by ERCP brushing, consistent with the scarcity of Base‐BEC in organoid scRNA‐seq data [41]. This further supports that VIM^+^ BEC originates from Base‐BEC. Previous BEC organoids established from whole biliary tissue exhibited EMT in vitro and in vivo, which may be attributed to the presence of PBG BEC [12]. MUC6 serves as a marker for PBG BEC (Base‐BEC) [26]. Furthermore, we isolated MUC6^+^ BEC to establish PBG BEC organoids and verified their EMT progression in vivo.

The transcription factor CREM displays relatively specific activity in VIM^+^ BEC, with both its expression levels and transcriptional activity significantly elevated during biliary fibrosis. The primary function of the CREM is to regulate the intracellular cAMP signaling pathway, which plays a crucial role in cellular differentiation, proliferation, and apoptosis [42]. CREM overexpression promotes the progression of atrial fibrosis [15, 16]. Our study revealed that the CREM expression level decreased during FR. Furthermore, CREM was found to be highly activated in VIM^+^ BEC and regulates VIM expression. Inhibition of CREM family activity can effectively suppress the progression of renal EMT and fibrosis, which was also proved by BEC‐specific Crem CKO rats in our LT models [17]. These findings indicate that CREM plays a crucial role in the phenotypic transition of BEC EMT by regulating VIM expression.

Subsequently, we found that extracellular factors induce the CREM expression in VIM^+^ BEC. The blood supply, cellular metabolism, and repair capacity of the bile duct are highly dependent on an adequate oxygen supply [43]. When local hypoxia occurs, the structure and function of the biliary system are prone to impairment, which directly induces or exacerbates NAS [14, 44]. The biliary tissues of NAS patients remain in a state of chronic hypoxia post‐LT [44]. Hypoxia promotes the expression of VEGF‐A and TGF‐β in BEC, induces the DR, and accelerates tissue fibrosis [45]. We found that the hypoxia and TGF‐β pathways were highly activated in the VIM^+^ BEC subcluster, and these pathways were downregulated during FR. Based on this, we established an in vitro BEC EMT model via hypoxia and TGF‐β stimulation, which significantly upregulated CREM and VIM expression and promoted the EMT process. We established an LT model with 30 min of warm ischemia to simulate the in vivo hypoxic process of grafts [2]. LT‐related graft fibrosis is accompanied by the upregulation of the hypoxia and TGF‐β pathway [44]. Imaging and pathological analyses showed similar features between post‐LT grafts in rats and clinical NAS patients, with enhanced progression of biliary EMT, fibrosis, and biliary tree stricture [2]. Meanwhile, we found that the expression of Crem and Vim in the biliary graft of rats was upregulated post‐LT. These findings suggested that hypoxia and TGF‐β may act as key extracellular factors to promote CREM expression in BEC.

Melatonin, a serotonin‐derived hormone primarily secreted by the pineal gland to regulate the sleep‐wake cycle [46], also shows high concentrations in the liver. Previous research indicates that melatonin can influence the expression of Icer in the rat pineal gland [18]. ICER acts as an early inhibitor of CREM, suppressing CREM expression during early transcription [19, 20]. Building upon this knowledge, we hypothesized that melatonin could be used to inhibit the expression of CREM in BEC post‐LT, thereby reducing the EMT in BEC and slowing down tissue fibrosis progression. And we verified the inhibitory effect of melatonin on CREM and VIM expression in BEC under hypoxia and TGF‑β stimulation, both in vitro and in vivo. Importantly, the clinical trial showed that some NAS patients who took melatonin exhibited a decreasing trend in liver function in the short term.

This study has several limitations. First, the imbalance in human scRNA‐seq sample groups limited the reliability of the translational conclusions. Second, in the clinical trial, some ALP and GGT levels did not recover to normal, while some patients showed no obvious efficacy or rebound post‐treatment, particularly for TBIL levels. This might be associated with the low dose (10 mg/day) used in our clinical trial, compared to 50 mg/(kg.day) in our animal experiments. 10 mg/day is a relatively low, well‐tolerated dose for long‐term administration in humans [47, 48]. Considering that LT recipients are in a special immunocompromised state, we chose a conservative and safe starting dose to preliminarily explore its biological effects on NAS and related molecular indicators. Third, as an exploratory study with only 7 enrolled participants and only one case with paired pre‐ and post‐melatonin treatment samples, the small sample size precluded robust statistical analysis and limited translational reliability. Thus, further large‐sample clinical studies with dose escalation are still needed to investigate the efficacy of melatonin and strengthen the clinical translational value.

In summary, we identified a novel EMT‐related BEC subcluster, VIM^+^ BEC, in the NAS‐related bile ducts. Under hypoxia and TGF‐β stimulation, CREM is activated in BEC, which promotes VIM expression in BEC and expansion of the VIM^+^ BEC subcluster, thereby driving tissue EMT and fibrosis. BEC‐specific Crem CKO suppressed the hypoxia/TGF‐β‐Crem‐Vim axis and ameliorated biliary EMT in rats post‐LT. Importantly, melatonin, a potential therapeutic agent, suppressed Crem and Vim expression, inhibited biliary EMT and fibrosis progression in rats post‐LT, and improved short‐term liver function in NAS patients. Our research provides new insights into graft biliary fibrosis in NAS patients and offers a novel potential therapeutic strategy to address the low success rate and prolonged treatment cycles of endoscopic treatment in NAS patients.

## Author Contributions

Zhaoyi Wu: Data Curation, Methodology, Formal Analysis, Resources, Visualization, Writing – Original Draft, Writing – Review & Editing. Nengsheng Fu: Data Curation, Methodology, Formal Analysis, Resources, Visualization. Zhongxin Huang: Data Curation, Methodology, Resources. Ensi Ma: Data Curation, Investigation, Resources. Yu Mou: Data Curation, Investigation. Xiaoshi Wang: Data Curation, Investigation. Tian Dong: Investigation. Yujun Zhang: Methodology. Danqing Liu: Data Curation. Di Jiang: Data Curation. Jingya Kuang: Data Curation. Rui Liao: Data Curation. Yifeng Tao: Conceptualization, Methodology, Supervision. Leida Zhang: Conceptualization, Methodology, Supervision, Writing – Original Draft, Writing – Review & Editing. Chengcheng Zhang: Conceptualization, Methodology, Project administration, Funding acquisition, Supervision, Writing – Original Draft, Writing – Review & Editing.

## Conflicts of Interest

The authors declare no conflicts of interest.

## Supporting information




**Supporting File**: advs75885‐sup‐0001‐SuppMat.docx.

## Data Availability

The scRNA‐seq data on bile ducts of NAS patients are being uploaded to the GSA database under accession code HRA024283. The data that support the findings of this study are available from the corresponding author upon reasonable request.
