# Supplementary material for: Melatonin Alleviates Graft Biliary Fibrosis by Inhibiting VIM+ Cholangiocyte Subcluster via Hypoxia/TGF‐β‐CREM‐VIM Axis
Source: Adv Sci (Weinh). 2026 Jun 3;13(47):e75885. doi: 10.1002/advs.75885 (PMC13336030; doi:10.1002/advs.75885)
Supplement: Supplementary file 1 [file ADVS-13-e75885-s001.docx]

Supporting Information

**Melatonin alleviates graft biliary fibrosis by inhibiting VIM⁺ cholangiocyte subcluster via Hypoxia/TGF-β-CREM-VIM axis**

Zhaoyi Wu^1^, Nengsheng Fu^1^, Zhongxin Huang^2^, Yu Mou^1^, Ensi Ma^3^, Xiaoshi Wang^1^, Tian Dong^1^, Yujun Zhang^1^, Danqing Liu^1^, Di Jiang^1^, Jingya Kuang^1^, Rui Liao^1^, Yifeng Tao^3,*^, Leida Zhang^1,*^, Chengcheng Zhang^1,*^

**Supplementary Materials and Methods**

**Definitions**

NAS was defined as strictures situated at a distance of no less than 5 mm proximal to the anastomosis. These strictures were identified using one of the following imaging modalities: T-tube cholangiography, magnetic resonance cholangiography, or ERCP.^[1]^ Endoscopic treatment success was defined as resolution of the stricture, confirmed by cholangiography performed at the end of the treatment protocol, along with relief of biliary obstruction symptoms, including pruritus and cholangitis.

**Tissue digestion and single cell collection**

Bile duct samples were rinsed twice with Dulbecco's modified Eagle’s medium (Gibco, NY, USA) and cut into 1 mm^3^ pieces. Next, 5 mL of a mixed digestion enzyme solution (0.03% collagenase type I, 0.05% collagenase type IV, 0.05% dispase, 0.01% DNase I) was added, and the tissues were incubated at 37°C for 50–70 minutes. The reaction was stopped with DMEM containing 10% fetal bovine serum (Gibco, NY, USA) and the sample was filtered through a 40 µm nylon mesh cell strainer. The precipitate was centrifuged at 350 x g for 5 minutes, and washed twice with DMEM containing 10% FBS. The cells were lysed with red Puregene RBC Lysis Solution (Qiagen, Duesseldorf, Germany) for 5 minutes at room temperature, and then dead cells and debris were removed with a Miltenyi Biotec Dead Cell Removal Kit (Miltenyi Biotec, Bergisch Gladbach, Germany). The live cell rate was assessed using trypan blue staining, with a minimum requirement of 85%.

**Hematoxylin and eosin (HE) staining**

The sample is fixed using formalin to preserve its structure. It is then dehydrated through a series of concentration gradient ethanol washes (75%, 85%, 90%, 100%) to remove water. The sample is embedded in paraffin to provide support for sectioning. Thin sections of the embedded sample are cut using a microtome and placed on glass slides. The sections are stained with hematoxylin and eosin to visualize structures such as cell nuclei and cytoplasm. The stained sections are then dehydrated and cleared again using xylene and different concentration gradient ethanol to prepare for mounting. The prepared slides are analyzed and interpreted under a microscope.

The extrahepatic bile duct histological alterations were systematically evaluated using standardized semi-quantitative scoring systems. The bile duct damage score (BDDS),^[2]^ which classified specimens into four progressive stages: grade 0 (normal epithelial architecture), grade 1 (epithelial flattening), grade 2 (epithelial destruction with intact subepithelial connective tissue), and grade 3 (complete structural disruption involving both epithelium and underlying connective tissue).

**Immunohistochemistry (IHC)**

Paraffin-embedded tissue sections (4 μm) were deparaffinized in xylene and rehydrated through graded ethanol. Antigen retrieval was performed by boiling sections in citrate buffer (pH 6.0) for 15 min. Endogenous peroxidase activity was blocked with 3% H₂O₂ for 10 min at room temperature. Sections were incubated with primary antibody against overnight at 4°C, followed by horseradish peroxidase-conjugated secondary antibody for 30 min at room temperature. Signals were visualized using 3,3'-diaminobenzidine substrates, and sections were counterstained with hematoxylin, dehydrated, and mounted.

**ScRNA-seq data processing**

We generated scRNA-seq profiles using 10x Genomics sequencing. The raw gene expression matrices from each sample were combined using CellRanger (version 6.1.2) with default parameters for demultiplexing, alignment to human reference genomes, and UMI counting (by using the cellranger count function). And subsequent analysis was performed in R (version 4.2.3) using Seurat (version 4.3.0) to analyze the gene-barcode matrices. SoupX (version 1.6.2) was used to estimate and eliminate cell-free mRNA contamination in droplet-based scRNA-seq data. All samples were merged into one Seurat object and filtered based on specific criteria (nFeature_RNA > 400, nFeature_RNA < 6000, percent of mitochondrial genes < 0.25 and hemoglobin genes < 0.03). Doublets were identified and removed using DoubletFinder (version 2.0.3) with the 92.5th percentile as the cutoff. A total of 151,509 cells were retained for further analysis.

**Clustering and dimension reduction**

We employed Seurat's "FindVariableFeatures" function to identify the 2000 most variable genes and performed PCA, cell clustering, and uniform manifold approximation and projection (UMAP) visualization to determine cell types. Harmony (version 0.1.1) was utilized to mitigate batch effects. The functions FindNeighbors in the Seurat package, with dimensions set to 1:30. And FindClusters, with a resolution set to 0.9, resulted in a total of 31 cell clusters. These clusters were accurately annotated into 10 major cell types using established marker genes. Subsequently, we characterized distinct cell types and subtypes within the samples.

For BEC subcluster analysis, we extracted all BEC from the integrated dataset and performed re-clustering using the same pipeline with parameters (resolution = 0.1). Highly variable genes were recalculated, and PCA, batch correction, UMAP visualization, and cell clustering were re-conducted to identify transcriptionally distinct BEC subclusters. Marker genes for each BEC subcluster were identified using the “FindAllMarkers” function, and subclusters were further defined and annotated according to specific molecular signatures.

**Assessment of the ratio of observed to expected cell number (Ro/e) for each cluster**

To evaluate the tissue distribution preference of each cell cluster, we calculated the ratio of observed to expected cell number (Ro/e). A cell cluster was defined as tissue-enriched when the Ro/e ratio was > 1.^[3]^ Heatmaps for visualizing the Ro/e distribution were generated using the R package pheatmap (version 1.0.12).

**Gene set variation analysis (GSVA)**

To comprehensively evaluate the pathway activity of individual cells and quantify the expression levels of genes involved in specific signaling pathways, we employed GSVA. The analysis was performed using the GSVA software package (version 1.46.0) in R, with default parameters unless otherwise specified. Specifically, we first downloaded the curated signaling pathway gene sets from the Molecular Signatures Database (MSigDB),^[4]^ focusing on hallmark pathways and canonical signaling pathways that are closely related to our research focus pathways. GSVA then calculated the enrichment score for each pathway in every individual cell by aggregating the expression levels of all genes within the corresponding gene set, thereby assigning a pathway activity estimate to each cell. This analytical approach enabled us to systematically compare pathway activity differences across distinct cell types and experimental groups, facilitating the identification of key signaling pathways that drive cellular functional differences in our study.

**Correlation matrix calculation**

The correlation matrix was visualized using heatmaps to identify clusters of co-expressed genes.

To evaluate cell-to-cell similarities, we first selected the top 1000 genes with the highest standard deviation of expression levels across all cell clusters for correlation analysis. Pairwise correlations between cellular gene expression profiles were calculated using Pearson correlation coefficients, which quantify the linear relationship between gene pairs. The resulting correlation matrix was visualized as a heatmap to identify clusters of co-expressed genes.

Furthermore, within the VIM^+^ EMT, we computed the correlation between CREM expression and the expression levels of all other genes. Genes positively correlated with CREM were visualized to illustrate coregulatory relationships.

**Analysis of single-cell trajectories**

Trajectory analysis was conducted with Monocle (version 2.24.0) to elucidate cell state transitions of BEC subclusters.^[5]^ Raw counts from targeted cell types were normalized using estimateSizeFactors and estimateDispersions functions with default parameters. DEGs for each subcluster were identified via FindAllMarkers function in Seurat, and were used to order cells in pseudotime. DEGs along pseudotime were determined with differentialGeneTest and visualized using plot_pseudotime_heatmap.

**Regulatory transcription factors analysis**

To systematically identify cell-type-specific gene regulatory networks (GRNs) and key transcription factors (TFs) driving cellular functional states, we performed SCENIC analysis using the R package SCENIC (version 1.1.2.2), following the standard analytical pipeline.^[6]^ The regulons and TF activities (AUCell) for each cell were calculated with motif collection version mc9nr. The analysis was conducted on the normalized scRNA-seq dataset of BEC subclusters.

The SCENIC analysis consisted of three core steps: first, we used the runGenie3 function to infer co-expression modules between TFs and their target genes, with default parameters to ensure robust module identification. Second, we applied the runSCENIC_1_coexNetwork2modules function to filter and refine the co-expression modules, retaining only those modules with significant TF-target gene regulatory relationships. Third, we used the runSCENIC_2_createRegulons function to construct regulons (TF-target gene networks) by integrating motif enrichment analysis, where only regulons with significant motif enrichment (top 5) were retained for downstream analysis.

Subsequently, we calculated the regulon activity score for each cell using the runSCENIC_3_scoreCells function, which quantifies the activity of each regulon in individual cells based on the expression levels of its target genes. Additionally, we focused on the regulons associated with CREM and VIM, analyzed their activity differences between experimental groups.

**Co-expression network analysis**

The R package hdWGCNA (version 0.3.00) is used for the co-expression network analysis. We then used the ConstructNetwork function with default parameters to construct the co-expression network, where the soft-thresholding power was automatically selected to ensure the network followed a scale-free topology. We used Cytoscape (Version 3.9.1) to map CREM co-expressed genes in VIM^+^ EMT.

**DEGs analysis**

We utilized Seurat's "FindMarkers" function to detect DEGs between the FP and the FR groups. A LogFC value and an adjusted P-value for each DEGs were calculated by the Wilcoxon rank-sum test. Genes with |avg_logFC| > 1 and p_val_adj < 0.01 were selected.

**Gene function analysis**

We utilized ClusterProfiler (version 4.6.2) to perform Gene Ontology (GO) enrichment analysis. and single-cell pathway analysis (SCPA) (version 1.6.1) to perform enrichment analysis, which helped us identify enriched biological processes and pathways associated with the different cell types and DEGs.^[7]^

**Analysis of public scRNA-seq datasets**

ScRNA-seq datasets derived from the Tabula Muris Senis dataset (GSE291336, GSE213452, and GSE239283) were retrieved to select BEC. All these datasets were analyzed by Seurat R package (version 4.2.3).

**Western blotting (WB)**

Tissues or cells simples underwent lysis using RIPA lysis buffer (Beyotime, Shanghai, China) supplemented with a protease inhibitor mix (Beyotime, Shanghai, China) for 30 minutes at 4 °C, followed by centrifugation at 13,000 g for 15 minutes at 4 °C. Protein concentrations were determined utilizing a BCA Protein Assay Kit (Beyotime, Shanghai, China). The proteins were subsequently denatured, separated by SDS-PAGE (Beyotime, Shanghai, China), and transferred onto NC membranes (GE Healthcare, UK). Following blocking with 5% skim milk, NC membranes were subjected to overnight incubation at 4 °C with specified primary antibodies. Subsequently, membranes were exposed to suitable secondary antibodies and scanned using a ChemiDoc imaging system (Bio-Rad, Hercules, CA, USA).

**Immunofluorescence (IF)**

Frozen sections were washed in PBS and fixed in 4% paraformaldehyde in PBS, and paraffin–embedded sections were deparaffinized and rehydrated. Antigen retrieval was performed, and the sections were permeabilized and blocked. The sections were incubated with primary antibodies overnight and then with secondary antibodies. The relevant antibodies and concentrations can be found in Table S1. The IF intensity was measured using ImageJ (v1.53t)^[8]^ and CellProfiler (4.2.6).^[9]^

**Reverse transcription polymerase chain reaction (RT-qPCR)**

Total RNA was extracted from tissue using an RNAiso Plus kit (TaKaRa, Code No. 9019) and reverse-transcribed into cDNA using a PrimeScript RT Reagent Kit with gDNA Eraser (TaKaRa, Code No. RR047A). Quantitative PCR was performed using a TB Green Premix Ex Taq II kit (TaKaRa, Code No. RR820A) on a CFX96 Real-Time PCR Detection System following the manufacturer's instructions. Each sample was tested in triplicate. The relevant human and rat primers can be found in Table S4.

**Cell delivery**

Biliary organoids were retrogradely delivered into the bile duct via the extrahepatic biliary tree. In brief, a fine-bore cannula was inserted and fixed into the gallbladder, and the distal common bile duct was clamped to direct the infusion toward the bile duct. The cell suspension was injected through the gallbladder cannula at a total volume of 1 μL/g body weight, with a maximum flow rate of 1 μL per second.

**ChIP-RNA**

Perform ChIP on 293T according to the ChIP Assay Kit protocol (Beyotime, Shanghai, China), followed by PCR analysis. Specifically, add formaldehyde to a final concentration of 1% in 10 mL cell culture medium and incubate at 37 °C for 15 min. Add 1.1 mL of Glycine Solution (10×), incubate at room temperature for 5 min, then aspirate the liquid. Wash twice with PBS containing PMSF. Add 1 mL of PBS containing PMSF to collect cells into a 1.5 mL EP tube. For every 1 × 10⁶ cells, add 200 µl SDS Lysis Buffer and sonicate on ice until DNA fragments are 400-800 bp. Take a portion of the supernatant as Input, then add an appropriate amount of Protein A+G to the remaining sample and slow-spin at 4 °C for 30 min. Subsequently, add the corresponding antibody and incubate overnight at 4 °C with gentle shaking. Add an appropriate amount of Protein A+G and incubate at 4 °C with gentle shaking for 1 h. After washing with different washing buffers, add Elution Buffer (1% SDS, 0.1 M NaHCO₃). Collect the supernatant, add 5 M NaCl, and heat at 65 °C for 4 h. Finally, proceed with PCR analysis. The ChIP-primers used in this study are showed in Table S4.

**Dual luciferase assay**

To investigate the potential binding of CREM to the VIM promoter, the pGL4-VIM-Promoter-WT and pGL4-VIM-Promoter-MUT reporter plasmids were synthesized by GenePharma (Shanghai, China). 293T cells (National Collection of Authenticated Cell Cultures) were co-transfected with the reporter plasmids together with pcDNA3.1-3×FLAG-CREM and the pGPL4-RL internal control plasmid using Lipofectamine® 3000 (Thermo Fisher Scientific, Waltham, USA). At 48 h post-transfection, luciferase activities were measured using the Dual-Luciferase Assay System (Thermo Fisher Scientific, Waltham, USA) following the manufacturer’s instructions. Renilla luciferase serving as an internal control.

**Flow cytometry analysis**

Tissue digestion and cell collection were conducted using the identical procedures employed in scRNA-seq. Cells were stained for 30 min at room temperature with antibodies (Table S3). Flow cytometry acquisition was performed in a FACSverse flow cytometer (BD Biosciences, CA, USA) and data were analyzed using FlowJo software.

**Hypoxia and TGF-β Stimulation** **Protocol**

When HEHBEC reached 70-80% confluence, the medium was replaced with serum-free 1640 medium for 12 h of starvation to synchronize cell status. Control group: Cells were cultured in serum-free 1640 medium under nonmonic conditions (37°C, 5% CO₂, 21% O₂) for the corresponding duration consistent with other groups. Hypoxia group: Cells were cultured in serum‑free RPMI 1640 medium under hypoxic conditions (37 °C, 5% CO₂, 1% O₂) for 1, 2, 4, 8, and 16 h, followed by 12 h of reoxygenation under normoxic conditions (37 °C, 5% CO₂, 21% O₂). TGF‑β group: Cells were incubated in serum‑free RPMI 1640 containing recombinant human TGF‑β1 (Sino Biological, Beijing, China) at 5, 10, 20, and 40 ng/mL under normoxic conditions for 24 h. Accordingly, 8 h of hypoxia followed by 12 h of reoxygenation was selected as the optimal hypoxic model condition, and 20 ng/mL TGF‑β1 stimulation for 24 h was chosen for the TGF‑β model.

**Rat LT**

Fast the rats overnight by removing all food in the cage, leaving ad libitum access to water. LT was performed according to the established cuff technique by as described.^[10, 11]^ LT of all groups underwent 30-min in situ warm ischemia. Rats were assigned to the following two groups: melatonin group received a single intraperitoneal injection of 50 mg/(kg.day) melatonin before transplantation, and intraperitoneally inject 50 mg melatonin every day after LT. CTRL group inject the same amount of saline. Euthanize the animals on days 7 and 14 post-LT.

**Serum Analysis**

The levels of ALT, AST, ALP, and TBA in rat serum were measured using assay kits (Rayto, Shenzhen, CN) on days 1, 3 and 7 (n = 5/group) after LT. Serum IL1β, IL6, IL10, and TNFα were measured using ELISA according to the manufacturer's instructions CUSABIO Life Sciences (College Park, MD, USA), following the manufacturer's protocols.

**Lentiviral Transfection**

To establish stable cell lines with CREM knockdown or overexpression, lentiviral vectors were used. These vectors were provided by Genechem (Shanghai, China). HEHBEC were seeded in 6-well plates (2×10⁵ cells/well) and cultured until 50-60% confluence. Lentivirus was added at a multiplicity of infection (MOI) of 10-20, along with 5 μg/mL polybrene to enhance transduction efficiency. After 24 h, the virus-containing medium was replaced with fresh complete medium. At 72 h post-transfection, cells were harvested for RT-qPCR to determine transfection efficiency. For stable transfection, cells were cultured in medium containing puromycin (2-5 μg/mL) 48 h post-transfection. The medium was refreshed every 2-3 days, and resistant colonies were formed after 7-10 days. Monoclonal cells were selected via limiting dilution, and CREM overexpression or knockdown efficiency was verified by RT-qPCR and WB.

**BEC-specific Crem CKO rats**

Crem^flox/flox^ rat model was developed by Shanghai Model Organisms Center, Inc. This model was generated by CRISPR/Cas9 system in SD rat background. Briefly, Cas9 mRNA was in vitro transcribed with mMESSAGE mMACHINE T7 Ultra Kit (Ambion, TX, USA) according to the manufacturer’s instructions, and subsequently purified using the MEGAclear^TM^ Kit (ThermoFisher, USA). A Crem donor vector containing flox sites flanking exon 5-6 of Crem gene were created. 5'-ACATTATCATTGGCCTAAACTGG-3' and 5'-GATTAAGTATAATAAGTCGCTGG-3' were chosen as Cas9 targeted guide RNAs (sgRNAs) and in vitro transcribed using the MEGAshortscript Kit (ThermoFisher, USA) and subsequently purified using MEGAclear^TM^ Kit. The donor vector with sgRNA and Cas9 mRNA was microinjected into SD fertilized eggs. F0 generation rats positive for homologous recombination were identified by long PCR. The primers (P1-P4) used for genotyping the correct homology recombination were P1: 5'-AAAATAGTTTTAGCTATCCTGGG-3' and P2: 5'-ACATTATCATTGGCCTAATAACTTC-3' for the correct 5' homology arm recombination, and P3: 5'-GACCCTGCAGGATAACTTCGT-3' and P4: 5'-TGGCCTAACATGCACAAGGT-3' for the correct 3' homology arm recombination. The positive F0 rats were chosen and crossed with SD rat to obtain F1 heterozygous Crem flox rats. Crem flox heterozygous rat were crossed with Sox9-Cre rat to generate Crem^flox/flox^; Sox9-Cre rats.

**RNA extraction, bulk RNA-seq data processing and analysis**

Total RNA was extracted from the tissue using TRIzol® Reagent in strict accordance with the manufacturer’s instructions. RNA quality was assessed with the Agilent 5300 Bioanalyzer, and concentration was quantified using the NanoDrop ND-2000 spectrophotometer. Only high-quality RNA samples meeting the following criteria were selected for sequencing library construction at Shanghai Majorbio Bio-pharm Technology Co., Ltd. (Shanghai, China): OD260/280 ratio of 1.8–2.2, OD260/230 ratio ≥ 2.0, RNA Quality Number (RQN) ≥ 6.5, 28S:18S rRNA ratio ≥ 1.0, and total yield > 1 μg.

RNA purification, reverse transcription, library construction, and sequencing were performed following the standard protocols provided by Illumina (San Diego, CA, USA). The RNA-seq transcriptome library was prepared using the Illumina® Stranded mRNA Prep Ligation Kit with 1 μg of total RNA. Briefly, messenger RNA (mRNA) was isolated via poly(A) selection using oligo(dT) beads and then fragmented with fragmentation buffer. Double-stranded cDNA was synthesized using the SuperScript Double-Stranded cDNA Synthesis Kit (Invitrogen, CA, USA) with Illumina random hexamer primers. The synthesized cDNA underwent end repair, phosphorylation, and adenylation (A-base addition) as per Illumina’s library construction guidelines. Target cDNA fragments of ~300 bp were size-selected on 2% Low Range Ultra Agarose gels, followed by PCR amplification with Phusion DNA polymerase (NEB) for 15 cycles. After quantification with the Qubit 4.0 Fluorometer, paired-end RNA-seq was performed on the DNBSEQ platform with a read length of 2 × 150 bp.

For DEGs identification between samples, transcript expression levels were calculated using the TPM method, and gene abundances were quantified with RSEM. Differential expression analysis was conducted using DESeq2, with DEGs defined as those meeting |log2 fold change (FC)| ≥ 1 and FDR ≤ 0.05. To explore the biological pathways associated with the DEGs, we performed Gene Set Enrichment Analysis (GSEA) using fgsea package (version 1.24.0) and MSigDB.

**Cholangiography**

A radiopaque silicone-based polymer compound with low viscosity and lead chromate composition (Flow Tech, Inc, Carver, MA) was administered via the common bile duct. Following common bile duct ligation, specimens were maintained at 4°C overnight prior to formalin fixation. Three-dimensional biliary tract reconstructions were subsequently performed using a high-resolution micro-computed tomography (Micro CT) scanner (Bruker, North Billerica, MA, USA).^[12]^

**Supplementary Figure Legends**

**Figure S1. The change of liver functions during fibrosis remission.**

(A) Bar plots showing the change of liver functions in NAS patients during fibrosis remission. 0, 1/4, 1/2, 3/4, 1 time represents the initial time point of treatment; one-fourth of the treatment duration; midpoint of treatment; three-fourths of the treatment duration; and endpoint of treatment, respectively.

**Figure S2. Quality control of seRNA-seq data from NAS patients.**

(A) Violin plots showing the quality control metrics of each sample.

(B) Bar plots showing the cell count of each sample.

(C) UMAP plot showing the overall distribution of all cells.

(D) UMAP plots showing marker genes for each cluster.

(E) Bar plot showing the cell type ratio of each sample.

(F) UMAP plot showing the overall distribution of each sample.

(G) Heatmap showing preferential enrichment of each cell type between the FP and FR groups.

(H) Bar plot showing the proportion of each cell type between the FP and FR groups.

**Figure S3. Changes in EMT potential during fibrosis remission.**

(A) UMAP plots showing the distribution of cell types between the FP and the FR group.

(B) UMAP plots showing the EMT scores of each cell types.

(C) Violin plots showing the EMT scores between two groups.

(D) IHC staining showing the expression of CDH1 and CDH2 in bile duct tissue of the two groups. And bar plots showing the CDH1 and CDH2 positive areas in the two groups. Scale bars, 50 μm.

**Figure S4. Subcluster analysis of BEC.**

(A) UMAP plots showing the BEC subclusters distribution of the FP and FR groups.

(B) Dot plot showing marker genes of each BEC subclusters.

(C) Heatmap showing preferential enrichment of each BEC subclusters between the FP and the FR group.

(D) IF staining of VIM and KRT7 in bile duct tissues from the two groups.

(E) UMAP plots showing the distribution of VIM expression in the two groups.

(F) UMAP plots showing the distribution of VIM and KRT7 expression in the BEC subclusters.

(G) Violin plots showing the VIM expression of VIM⁺ BEC between two groups.

(H) Pseudo-trajectory of five BEC subclusters.

(I) Expression dynamics of KRT19, SOX9, VIM, and CREM throughout the Pseudo-trajectory.

**Figure S5. Integrated scRNA-seq analysis of NAS, CCA and BEC organoid data.**

(A) UMAP showing the distribution of BEC subclusters.

(B) Dot plots showing the characteristic genes of BEC subclusters.

(C) Bar plots showing the proportions of Base-BEC and VIM⁺ BEC among the three groups.

**Figure S6. Construction of biliary organoids.**

(A) Bright-field images of biliary organoid. Scale bars, 500 μm.

(B) IF showing the expression of CK7, MUC6 and VIM in biliary organoids from the sorted and non-sorted groups. Scale bars, 500 μm.

**Figure S7. Correlation analysis between CREM and EMT-related genes.**

(A) Dot plot showing the correlation between genes.

(B) UMAP plots showing the distribution of VIM, COL3A1 and CREM expression in the BEC subclusters.

**Figure S8.** **Biliary fibrosis is accompanied by elevated EMT progression and CREM expression.**

(A) Dot plot showing the GO enrichment pathway of various BEC subclusters.

(B) Violin plots showing the EMT related genes expression between the FP and FR groups.

(C) Co-expression network of the CREM gene in the VIM^+^ BEC.

(D) WB analysis of CDH1, CDH2 and VIM protein levels in HEHBEC after exposure to hypoxia for 0, 1, 2, 4, 8 and 16 h, or treatment with TGF‑β at concentrations of 0, 5, 10, 20 and 40 ng/mL.

(E) IF staining with phalloidin to observe morphological changes of HEHBEC after hypoxia and TGF-β stimulation.

**Figure S9. Biliary grafts in post-LT rats develop fibrosis.**

(A) Bar plots showing liver function (ALT, AST, ALP, ALB, and TBA) levels in rats at 1, 3, 7, 14 and 28 days post-LT.

(B) Bar plots showing serum levels of IL‑1β, IL‑6, IL‑10 and TNF‑α in rats at 1, 3 and 7 days post-LT.

(C) Violin plots showing the changes in EMT pathway scores and the expression of Crem at 1, 3, and 7 days post-LT of rats scRNA-seq data.

(D) Violin plots showing the hypoxia and TGF-β signaling scores at 1, 3, and 7 days post-LT of rats scRNA-seq data.

(E) Bar plots showing the Cdh1, Cdh2, Vim, and Crem positive areas of bile ducts in each group.

(F) IF staining of Vim and Crem in bile duct tissues at 7 and 28 days post-LT in rats (left). Bar plot showing the mean fluorescence intensity of each group (right). Scale bars, 200 μm

**Figure S10.** **CREM knockdown in BEC in vitro and in vivo.**

(A) Relative mRNA expression of CREM in HEHBEC after CREM overexpression and knockdown via lentiviral transfection.

(B) Protein expression levels of CREM in HEHBEC after CREM overexpression and knockdown via lentiviral transfection.

(C) Electrophoresis bands from DNA detection of rats’ tail tissue.

**Figure S11.** **Pathological staining of bile duct tissues from CKO and CTRL groups post-LT.**

(A) IF showing the expression of Ck7, Vim, and Dapi in the CTRL and CKO groups. Scale bars, 500 μm.

(B) Crem, Cdh1, Cdh2, Vim, α-SMA, Masson, and Sirius red staining of bile ducts in the sham, CTRL, and CKO groups. Bar plots showing the positive areas of bile ducts in each group. Scale bars, 100 μm.

(C) PCA score plot showing the distribution of transcriptomic data of the CTRL and CKO groups (n=3 per group).

(D) Bar plot showing the Vim gene counts between two groups.

**Figure S12.** **Liver function and IF staining results in CTRL and melatonin groups**

(A) Bar plots showing the Masson, Sirius red, α-SMA, Cdh1, Cdh2, Vim, and Crem positive areas of bile ducts in the CTRL and melatonin groups.

(B) IF showing the expression of Ck7, Vim, and Dapi in the CTRL and melatonin groups. Scale bars, 500 μm.

(C) Bar plots showing the liver function in the CTRLand melatonin groups.

(D IF showing the expression of CREM in the CTRL and melatonin groups. Scale bars, 500 μm.

**Figure S13.** **Safety assessment of oral melatonin administration in NAS patients.**

(A) Bar plots showing the PSQI and TESS scores of each patient at 0, 4, and 8 weeks.

**Supplementary Figures**

**Suppl Figure 1**

**Suppl Figure 2**

**Suppl Figure 3**

**Suppl Figure 4**

**Suppl Figure 5**

**Suppl Figure 6**

**Suppl Figure 7**

**Suppl Figure 8**

**Suppl Figure 9**

**Suppl Figure 10**

**Suppl Figure 11**

**Suppl Figure 12**

**Suppl Figure 13**

**Supplementary Tables**

**Table S1.** Comparisons of NAS patients’ baseline characteristics between FP and FR groups.

|  | FP (n=18) | FR (n=4) | *P* value |
| --- | --- | --- | --- |
| Donor age ^a^ | 44.00 [33.50, 51.75] | 49.00 [45.00, 54.00] | 0.765 |
| Donor sex (Male) ^b^ | 4 (100.0) | 12 (85.7) | 1 |
| Donor BMI ^c^ | 22.10 (0.68) | 24.42 (3.48) | 0.209 |
| DBD ^b^ | 1 (25.0) | 9 (75.0) | 0.233 |
| Age ^c^ | 54.50 (4.80) | 47.39 (13.96) | 0.334 |
| Sex (Male) ^b^ | 4 (100.0) | 18 (100.0) | - |
| BMI ^a^ | 29.00 [26.17, 29.73] | 22.30 [21.10, 26.80] | 0.147 |
| MELD scores ^c^ | 18.75 (13.45) | 18.44 (8.09) | 0.952 |
| Child Pugh ^b^ |  |  | 0.4 |
| A | 2 (50.0) | 6 (33.3) |  |
| B | 0 (0.0) | 6 (33.3) |  |
| C | 2 (50.0) | 6 (33.3) |  |
| CIT ^c^ | 373.50 (77.43) | 382.11 (99.34) | 0.873 |
| HAT ^b^ | 0 (0.0) | 2 (14.3) | 1 |
| Severity of NAS ^b^ |  |  | 0.029 |
| None |  | 4 |  |
| Mild | 8 |  |  |
| Moderate | 6 |  |  |
| Severe | 4 |  |  |
| Time from LT to NAS (Day) ^a^ | 174.00 [114.00, 285.25] | 472.00 [334.50, 566.50] | 0.021 |
| Endoscopic treatment duration (Day) ^a^ | 451.50 [117.50, 736.00] | 1358.00 [791.75, 1927.25] | 0.027 |

BMI, body mass index; DBD, donation after circulatory death; MELD, model for end-stage liver disease; CIT, cold ischemia time; HAT, hepatic artery thrombosis; NAS, non-anastomotic stricture.

^a^ Values are median (interquartile range)

^b^ Values are count (percentage)

^c^ Values are mean (SD)

**Table S2.** Baseline characteristics of NAS patients enrolled in the melatonin clinical trial.

|  | Overall (n=7) |
| --- | --- |
| Age (mean (SD)) | 44.86 (9.89) |
| Sex (Male) (%) | 2 (28.6) |
| BMI (mean (SD)) | 20.98 (2.98) |
| Time from LT to NAS (Day) (median [IQR]) | 196.00 [138.00, 312.50] |
| Endoscopic treatment duration (Day) (mean (SD)) | 983.14 (741.73) |

**Table S3. Antibodies used for IF, WB, and flow cytometry (FC).**

| Name | Cat no. | Host | Supplier | Assay |
| --- | --- | --- | --- | --- |
| CDH1 | ab231303 | Rabbit | abcam | IF,,IHC,WB |
| CDH2 | ab18203 | Rabbit | abcam | IF,IHC,WB |
| CREM | sc-390426 | Mouse | santa | IF,IHC,WB |
| VIM | AF300187 | Rabbit | AiFang biological | IF,IHC,WB |
| CK7 | ab181598 | Rabbit | abcam | IF |
| MUC6 | ab216017 | Mouse | abcam | IF |
| KU80 | MA5-32212 | Rabbit | Invitrogen | IF |
| α-SMA | H641505024 | Rabbit | HUABIO | IHC |
| ICER | ab64832 | Rabbit | abcam | WB |
| β-actin | 66009-1 | Rabbit | proteintech | WB |
| CDH1 | 20874-1-AP-50 | Rabbit | proteintech | FC |
| CDH1 | sc-59778 | Rat | santa | FC |
| CDH2 | NBP1-48309AF594 | Mouse | Novus | FC |

**Table S4. Primers used for RT-qPCR.**

| Gene | Organism | Forward | Reverse |
| --- | --- | --- | --- |
| CREM | Human | AGTCCCTGCCCTTTGTACACA | GATCCGAGGGCCTCACTAAAC |
| Crem | Rat | AGTCCCCAGCAACTAGCAGA | CACAGTCAACCAGGTCCAA |
| Vim | Rat | CTGGTTGACACCCACTCCAA | AAGGTCATCGTGGTGCTGAG |
| Crem-flox | Rat | GCTGAGTGTGCATGATAAGTGG | ACTGCAGCACAAATTAACAAAGT |
| Crem-ko | Rat | AGCATTCTTCTTCGGACCTATCT | ACTGCAGCACAAATTAACAAAGT |
| Sox9-2A-Cre | Rat | P2- AAGTCCAAACAGGCAGGGAG | P1-TCACCTACATGAACCCAGCG P3- ATCAGCCACACCAGACACAGAGATC |
| VIM-site1 | Human | AAGAGAGCAGCCTATCACAGC | TGGTCCTCTTCATCATCTCAGG |
| VIM-site2 | Human | AAGAGGACCAGTGCCCATTC | GCTTGCTGACTGACTCACTATG |
| VIM-site3 | Human | CAATGCCTTGTCCTCCTT TTCC | TGCCTGGAACCCTTAGAATGAG |

**Referrence**

1.	Buis, C.I., et al., *Nonanastomotic biliary strictures after liver transplantation, part 1: Radiological features and risk factors for early vs. late presentation.* Liver Transpl, 2007. **13**(5): p. 708-18.

2.	Brunner, S.M., et al., *Bile duct damage after cold storage of deceased donor livers predicts biliary complications after liver transplantation.* J Hepatol, 2013. **58**(6): p. 1133-9.

3.	Guo, X., et al., *Global characterization of T cells in non-small-cell lung cancer by single-cell sequencing.* Nat Med, 2018. **24**(7): p. 978-985.

4.	Subramanian, A., et al., *Gene set enrichment analysis: a knowledge-based approach for interpreting genome-wide expression profiles.* Proc Natl Acad Sci U S A, 2005. **102**(43): p. 15545-50.

5.	Qiu, X., et al., *Reversed graph embedding resolves complex single-cell trajectories.* Nat Methods, 2017. **14**(10): p. 979-982.

6.	Aibar, S., et al., *SCENIC: single-cell regulatory network inference and clustering.* Nature Methods, 2017. **14**(11): p. 1083-1086.

7.	Wu, T., et al., *clusterProfiler 4.0: A universal enrichment tool for interpreting omics data.* Innovation (Camb), 2021. **2**(3): p. 100141.

8.	Schneider, C.A., W.S. Rasband, and K.W. Eliceiri, *NIH Image to ImageJ: 25 years of image analysis.* Nat Methods, 2012. **9**(7): p. 671-5.

9.	Carpenter, A.E., et al., *CellProfiler: image analysis software for identifying and quantifying cell phenotypes.* Genome Biol, 2006. **7**(10): p. R100.

10.	Kobayashi, E., et al., *Protocol for the technique of orthotopic liver transplantation in the rat.* Microsurgery, 1993. **14**(8): p. 541-6.

11.	Wu, Z.Y., et al., *Proximal splenic artery ligation improves liver reperfusion, mitigates graft injury and decreases complications after liver transplantation.* Hepatobiliary Pancreat Dis Int, 2026.

12.	Masyuk, T.V., E.L. Ritman, and N.F. LaRusso, *Quantitative assessment of the rat intrahepatic biliary system by three-dimensional reconstruction.* Am J Pathol, 2001. **158**(6): p. 2079-88.
